# Gynæcological Society of Chicago—Regular Meeting, June 29

**Published:** 1888-10

**Authors:** 


### TRANSACTIONS OF THE GYNÆCOLOGICAL SOCIETY OF CHICAGO.

The Regular Meeting was held on Friday, June 29, 1888, the President, HENRY T. BYFORD, M. D., in the chair.

Dr. DANIEL T. NELSON presented a "Sarcoma of the Ovary with half-twisted Pedicle, removed by autopsy."

He said: I have a specimen here, the interesting points of which will be brought out in the history. This was a post-mortem operation, but it demonstrates, I think, that sometimes surgical interference may be the better course when the patient's condition is nearly or quite hopeless. It is better for us at least to make an exploratory incision and arrive at a clearer diagnosis, which will possibly enable us to do something for the life of the patient, than to pursue an expectant course. It would seem as if this patient might have been saved had an operation been attempted early enough; probably she was not seen by any physician in regular attendance early enough to have insured her life by operation, still there will always be a doubt in regard to that.

I first saw the patient in consultation several days before she was taken to the hospital. She was a patient of Dr. J. E. De Wolf, of Englewood, whom I invited to be present to-night, but, unfortunately, he had a professional engagement. She was taken to the Woman's Hospital, but operation was delayed from one day to another, waiting for her to improve in condition, which she never did, and we have the tumor here by post-mortem removal. Her history is very scant, and yet some points in it are of interest, and will raise queries that I trust some of you will be able to answer.

Mrs. M. entered the Woman's Hospital June 7, 1888; occupation, housewife for many years; age at puberty, twelve; age on entering the hospital, thirty-nine. She was born in America of French and German parents, had been twice married; the first time seven months, the second time seventeen years. She was the mother of nine children, one by her first husband and eight by her second. After the birth of her last child, seven years ago, she did not menstruate for four years; since that time there have been irregular menstrual periods. It is so stated in the history, and yet I think we should rather say there were hæmorrhages from the uterus during these past four years. One year ago she noticed a fluid discharge from the rectum. This is a nice question in pathology, to my mind. She gave evidence of some inflammatory process in the right ovarian region—tenderness, soreness, some elevation of temperature, was confined to bed for a time, and there was a sudden discharge of a considerable quantity of blood. Such quantities are never rightly estimated, but the amount was guessed at by the patient at more than a pint, and supposed by her to have passed from the rectum. Perhaps that was not correct; at all events, after that bloody discharge, she was relieved of the swelling, the tenderness, the inflammatory process, whatever it was, and resumed her ordinary duties. Some time afterward, but unfortunately the record does not say how soon afterward, she began to suffer from swelling in the same region, that continued up to her death. There was constant soreness in the right inguinal region; three months ago the abdomen began to enlarge and she to gain in flesh, strength, and vigor, so that her attending physician, without making a local examination, and especially her neighbors, supposed she was pregnant. She felt comparatively well until four weeks previous to entering the hospital, when she began to suffer severe pain, tenderness in the right inguinal region, and there was evidence of some kind of tumor. On going to bed with her last illness, about a week before she entered the hospital, her physician became satisfied that there was something more than pregnancy, that there was inflammation of some type. Some days before she entered the hospital I saw her in consultation, and advised a removal to the hospital in the hope that there might be some kind of an operation for her relief. On entering, her temperature was 100 2-5° Fahr., pulse 104. The following day the temperature was 100 4-5° Fahr., pulse 132; the following afternoon the pulse was 132, temperature 100° Fahr. and a fraction. On the morning of the fourth day the temperature ran down to 99½° Fahr., and the pulse to 119. Possibly an operation might have been performed then, and her life saved, but a more convenient and better time was sought for, that never came. There were the usual evidences of peritonitis, and death in the usual way followed. When she first entered the hospital her bowels were moved, but not afterward; vomiting came on the third day, but passed off on the fourth, when probably an operation could have been performed with the possibility of saving life. She died on the sixth day after entering the hospital, and a few hours after death a post-mortem examination was made and this tumor removed. The appearance is somewhat changed now, but yet it presents fairly well the appearance at the time of the autopsy. You notice the dark, venous, congested appearance of a portion of the tumor. This was the anterior portion as it presented against the abdominal wall, very slightly adherent; no adhesions from old inflammation, either to omentum or other structures, but *a half-twisted pedicle.* The pedicle has been tied in such a way as to retain that appearance as much as possible. Here we have the broad ligament that is simply half twisted and tied in that position on purpose. The evidence of completely twisted pedicle and death of the tumor were not present. There was simply an increase in size resulting from the congestion, but no sloughing, no death of the part; a slow, inflammatory process had taken place in the tumor and subsequently in the peritoneum that was the cause of death. The obstruction of the bowels, I believe, was due to the peritonitis, and not to pressure from the tumor. It has not been my privilege to see a patient with a tumor and twisted pedicle, but it seems to me I could have recognized it; but this being only half-twisted, the circulation was impeded, not stopped. The tumor has been examined by Dr. Frank Carey, and the report is sarcoma. There was, so far as I saw, and I made a rather hurried examination, no evidence of the disease extending to other organs; there is no evidence of it in the pedicle; there was no evidence in the glands or intestines or other structures adjacent; so it seems as if it could have been entirely removed if the operation had been performed during the life of the patient. The uterus was a little enlarged, but no other evidence of disease about it. I made a diagnosis of malignant tumor, without being exactly certain as to its nature, but it seemed to me malignant on account of its rapid development and the age of the patient. I did not regard it as a uterine tumor, as the uterus was movable and the tumor seemed to be separate from it. Within the abdominal walls there was a considerable amount of ascitic fluid, so that the abdomen was very tense, and it was difficult to say whether or no the tumor could be moved readily within the abdominal walls. I was unable to say whether or no there were adhesions, but from the ascites I hoped not.

Professor CHRISTIAN FENGER presented the following specimens:

FIBRO-CYSTO-SARCOMA OF THE UTERUS.

This specimen was removed by laparotomy from a woman of thirty-five, who had a tumor the size of a child's head, immovably connected with the uterus at the fundus, and also two small myomas that could be felt through the vagina. The large tumor showed fluctuating places on the surface, by palpation through the abdominal wall, and I concluded that it was an ovarian cystoma, either located in the broad ligament or sufficiently adherent to the uterus to make them move together. At the operation I found it to be a cysto-fibroma, or fibro-cysto-sarcoma, subperitoneal, but attached by the broad base to the uterus at the fundus. After temporary elastic constriction around the cervix, the tumors were enucleated, and as the uterine cavity was not opened, I united the wound of the wall of the uterus with buried step sutures, deep and superficial, and a final continuous suture along the inverted borders of the peritoneum.

At the close of the operation all hæmorrhage had apparently stopped, consequently I did not drain. In the course of the first week some fever set in, and on the tenth day I reopened the lower border of the wound, and evacuated about three to four ounces of blood, mixed with pus, from a cavity surrounding the body of the uterus. The evacuation and subsequent washing out and drainage did not have much influence on the patient's condition. The fever continued; she had a large gangrenous bedsore over the os sacrum, and died six days later, in the third week after the operation. The autopsy showed no peritonitis, and the cavity, with the accumulation of blood and pus, was found entirely separate from the general peritoneal cavity. On examining the uterus, I found, as you see here, surrounding the line of the uterine wound, an island of gangrenous tissue including the wound and a square inch or more to each side. This gangrene explains the persistence of fever and sepsis, notwithstanding the evacuation and drainage.

The large tumor has, you see, a smooth surface. On the cut surface, in some parts, there was an appearance of myoma; in other places, islands of softer tissue, looking like myxoma or sarcoma, and in other parts, cystic cavities. These cysts have not the usual shape and appearance of cystomas, but are irregular, triangular, or longitudinal sinuses, the walls of which are not smooth, but trabeculated, so as to give the appearance, as Dupuytren describes it, "similar to the walls of the ventricles of the heart."

I shall here make a few remarks on fibro-cystomata of the uterus, because they are comparatively rare, the whole number described in the literature not being much above one hundred. Fibro-cystomata are, as the name indicates, forms of fibromata, or myomata, and it is a comparatively rare change in the pre-existing elements of these tumors that gives them the additional characteristics of cystomata.

We distinguish between the following varieties: Myxo-myoma, as described by Virchow, characterized by œdema of the interstitial tissue, and by the fluid in the spaces containing mucin; consequently it is something more than a simple œdema of the myoma. Spread islands of embryonal cells are also proof of a more active process, terminating in myxomatous, or even sarcomatous, tissue. Besides the œdema in the interstitial tissue of the myoma, we find œdema and atrophy of the muscular fibres, isolated fibres, or their débris mixed with the fluid in the cavities. These cavities are of all sizes, from the microscopic, as shown on this slide, up to the size of a pin's head or walnut, and we even find cavities of enormous size containing several quarts of fluid. The cavities are lined with pavement-celled epithelium, or rather endothelium, as you would expect, since they originate from dilated lymph spaces, or naked when the cavity is formed by the disintegration of muscular fibres. The cavities contain clear, colorless, or bloody fluid that often coagulates spontaneously when evacuated—a fact that Atlee pointed out as a differential diagnostic sign in contradistinction to the fluid from ovarian cystomas. A special form is described as fibro-myoma lymphangiectodes, by Leopold. Distinctly different from this is the myoma teleangiectodes sive cavernosum of Virchow, with multiple cavities from the size of a millet-seed to that of a pea, communicating with the blood-vessels, and consequently containing pure blood. These tumors are found to enlarge during menstruation (Virchow), and on auscultation a bruit is heard (Péan).

As to the place of development, the great majority are subperitoneal. Of the seventy cases gathered from the literature by Heer, sixty-three were subserous, five interstitial and only two submucous tumors. They sometimes attain an enormous size, weighing twenty-nine, forty, and in one instance even eighty-one pounds.

The cysto-fibromata are most often found between the ages of 30 and 50. The symptoms are in the main, of course, the same as those of common myomata and fibromata. Uterine hæmorrhage is rare because, as before mentioned, they rarely develop close to the mucous membrane. A more characteristic symptom is a sudden enlargement, probably from acute increase in the size of the cysts or from intracystic hæmorrhage. The spontaneous coagulation of the fluid would be a valuable symptom if it was constantly found, but in about seventy cases it was noted in only eleven (Heer). It might, however, in reality, be more frequent, since in a number of cases it might not have been noticed (Gusserow). The lack of vitality shown by the tendency to local gangrene is also somewhat characteristic of these tumors. Thus Grammaticati, as stated by Gusserow, saw a myoma the size of a child's head, located in the wall of the cervix, undergo superficial necrosis, followed by sepsis and death.

It is rather noteworthy that a correct diagnosis was rarely made. They were almost always taken for ovarian cystomas, and a number of them were punctured. Puncture, however,in this form of cystoma is far more dangerous than in other cystomas, as shown by Leopold, who found that, as a consequence of puncture, ten patients out of eleven died. McGuire, therefore, is right in asserting that exploratory laparotomy is less dangerous than puncture.

The treatment should be early extirpation, because of the probability of rapid enlargement, the danger of puncture, the liability to gangrenous or septic changes, and thrombosis of the vessels in and around the tumor. Gusserow gives a series of forty-one laparotomies with twenty-two recoveries, the cause of the high mortality being the necessity of the removal of the uterus in some of the cases. Occasionally the operation cannot be finished; thus, according to Gusserow, in thirty-eight cases, seven were unfinished, and of the seven, six patients died. That an exact diagnosis, with a definite premeditated plan of operation, is of extreme importance, is shown by Gusserow, who out of eleven cases described in the literature, reported nine recoveries.

A few words about uterine sarcomata, inasmuch as the tumor here presented is a mixed form of cysto-fibroma and sarcoma. In the uterus we distinguish between circumscribed and diffuse sarcomas, the former originating in the muscular wall of the uterus, the latter in the mucous membrane. The circumscribed uterine sarcomas are of the most interest to us in this connection, as they stand in near relationship to fibromyomas and fibro-cystomas. They form, usually, round, circumscribed, harder or softer tumors, looking like, and developing in the same places as, the fibro-myomas, and so similar to these that we must class the relapsing fibromas of Paget among the sarcomas. But besides more or less typical fibrous or muscular cells, here we find islands of short, spindle-shaped, round or polymorphous cells, or islands of myxoma tissue; in general, a more vivid cell-formation than in fibromas and myomas; and we further find in the same tumor in different places different forms of cells. So predominating, however, are fibroma or myoma tissue cells that Schröder regards it as a law that the circumscribed sarcomas are always formed by transformation of fibromas. According to Gusserow, the transformation of fibromas into the mixed form of fibro-sarcomas, myxo-sarcomas, and cysto-sarcomas is so rare that the literature shows very few well-observed cases of this kind. By examining the microscopic slides that I exhibit to-night, we find, in some portions, apparently typical myo-fibroma tissue, without or with dilated lymph spaces, in which we find granulated matter containing loose or isolated muscular cells; in other places, islands of typical myxoma tissue, here and there islands of embryonal cells; in another part of the tumor, territories of short, spindle-shaped cells, large and with oval or round nuclei; in other words, islands of unmistakable sarcoma tissue; and finally, places of common typical, round-celled sarcoma tissue.

As to the age in which fibro-sarcomas of the uterus are found, there is this difference from the cysto-fibromas, that although they both are most common betwen the ages of 30 and 50, the sarcomas are still common between 50 and 60, while the cysto-fibromas, as we have seen, stop at the age of 50.

As regards treatment, the sarcoma is a malignant tumor, and needs more extensive removal or radical treatment than the benignant cysto-fibroma. The removal of subserous or interstitial fibro-sarcomas by abdominal supra-vaginal extirpation and extra-peritoneal treatment has often been followed by a growth of sarcomatous tissue in the cicatrix in the abdominal wall. The abdominal total extirpation of the uterus can hardly be said to have lost much of its dreadful mortality of about 70 per cent. from the time of Freund's first operation till now.

In the treatment of this case the following suggestion occurred to me—a suggestion which was not carried out because of the patient's death. I should operate as I did, enucleating the subserous tumor, and if the uterine cavity was not opened, try intra-peritoneal treatment of the stump. After recovery from this operation, if the microscopic examination of the tumor proved it to be a fibro-sarcoma, I should follow, as soon as the patient's strength would permit, by vaginal extirpation. In the rare cases in which the size of a diagnosed circumscribed uterine sarcoma or fibro-cystoma will permit of vaginal extirpation, this operation is, of course, the only one indicated.

The two other specimens are not strictly gynæcological, as they occurred in men. However, they had this in common with gynæcology, that laparotomy had to be made.

COLLOID CARCINOMA OF THE CÆCUM.

This specimen is a tumor of the cæcum, a so-called colloid carcinoma. The patient was a man of about forty, in whom, for about six months, an increasing tumor had developed in the middle of the abdominal cavity. When I saw him, the tumor was of the size of the head of a child of four, was somewhat movable from side to side, and up and down. There were never any disturbances from the side of the intestines, but emaciation and considerable pain. I thought it a tumor of the omentum on account of its mobility, also that it was malignant because it was hard, nodular, and of rapid growth, but I did not think of the intestine being the seat because there were no symptoms. When the abdominal cavity was opened, I found this large nodulated tumor with a great many adhesions to the omentum and some to the intestines, and finally having separated these and applied a great many ligatures, when I got the tumor isolated and out through the abdominal wound, I found the ileum passing into one side of the tumor and the ascending colon coming out of the other side. I then divided the ileum and ascending colon two inches away from the tumor, detached and ligated the mesentery, and after the removal of the tumor closed the ileum and ascending colon in the usual way by invagination and suture, and made an anastomosis between the lower end of the ileum and upper end of the ascending colon by means of Senn's decalcified bone plates. The territory of approximation was covered by an undetached omental flap. I preferred this operation to circular resection or implantation of the ileum into the colon, because of the shortness of the plate operation as compared with the others. The patient lived four days, was able to take some liquid nourishment, had no vomiting, no tympanites, showed no symptoms of sepsis or peritonitis, but gradually became weaker and died. The autopsy showed no peritonitis; the ends of the upper and lower bowel were closed, as you see in this specimen, the closed ileum and closed end of the ascending column, and at a distance of two and a half inches the anastomosis covered with the omental flap which did not adhere. The peritoneal surfaces between the plates are perfectly united, allowing of no escape of liquid or air. The passage between the ileum and colon is perfectly free, as you see after opening the opposite wall of the intestines. The tumor shows at this point the ileum entering the large irregular cavity containing some liquid fæces, slightly tinged with blood, and at the upper end of the cavity is the ascending colon. This enormously thickened wall of the cavity, one and a half to two inches in thickness, is the carcinomatous intestinal wall, the cut surface presenting the characteristic gelatinous appearance of colloid carcinoma. This form of carcinoma has as its characteristics, in distinction from other carcinomas, colloid degeneration of the cells, causing them to enlarge, meet together, and form this transparent gelatinous substance. While we do not recognize a colloid carcinoma as distinctly different from carcinomas in general, as we know that partial colloid degeneration is common in all carcinomas of the intestinal tract, clinically, we recognize the extremes of this degeneration as a distinct form, characterized by its enormous size, and not uncommon in the stomach, large intestine, and peritoneum. In the peritoneal cavity there were no secondary tumors nor were the lymph glands of the mesentery invaded. This is what we should expect, as this colloid carcinoma is, as a rule, relatively benignant, with little tendency to the invasion of distant tissues or organs.

The death of the patient I ascribe to the fact that when the vitality has been lowered to a certain point by malignant tumors, without or with functional disturbances of vital organs, the organism loses its power to withstand more than a certain amount of operating, and death will follow from the yet unexplained exhaustion in spite of the absence of all the common well-known fatal complications.

DOUBLE CARCINOMA OF THE COLON.

The third and last specimen is from a man between forty and fifty, who had suffered terribly from difficult passages from the bowels for a number of months. Finally a small, almost immovable, tumor appeared to the right of the umbilcus, and later on distention of the small intestines, with pain and vomiting. Every half-hour or hour there would be a paroxysm of peristaltic contractions with excruciating pain. He finally asked to be relieved at any risk. On account of his extreme emaciation and weakened condition, I thought it out of the question to attempt extirpation, and resolved to try to relieve him by means of anastosmosis between the intestine above and below the stricture. Laparotomy revealed the tumor to be a carcinoma of the ascending colon ; consequently I united the lower part of the distended ileum with the empty transverse colon five or six inches away from the tumor. The patient did not get much relief and died ten days after the operation, growing gradually weaker, as in the other case. The autopsy showed no peritonitis, the omental flap was partially adherent to the intestine, the peritoneum between the plates united, but at the distal end of the plate, in the colon, an island of necrosis of the intestinal wall from pressure-atrophy caused by the plate. Thus, in this case, perforation of the intestine was only a question of a short time. The carcinoma of the ascending colon, as the specimen shows, is three inches long and has caused almost comple occlusion of the bowel. The reason why no relief followed the operation was found below the anastosmosis in the splenic flexure of the colon where a second carcinoma had developed, causing, as you see, almost complete obstruction of the colon. This second carcinoma was not discovered during the operation, as it was hidden high up under the spleen. The emptiness of the transverse colon, together with the rarity of a second carcinoma, was the cause of my not suspecting its presence. If it had been discovered, the anastosmosis would have been made between the ileum and the sigmoid flexure, of course. The mortality from even palliative operations upon the intestines is large, because, as a rule, the patients do not come to us for operation until they are exhausted by serious intestinal disturbances, usually of long continuance. This is so generally the case, that collapse, even after a short operation, is of frequent occurrence.

Senn's operation of intestinal anastosmosis with the plates does not take any more time than the abdominal operation for artificial anus. The last operation here mentioned was of thirty-eight minutes duration, from the time of the incision in the abdomen to the dressing of the abdominal wound.

The PRESIDENT: I intended to exhibit, for Dr. William H. Byford, a uterine cystomyoma possessing all the characteristics of the one just presented by Dr. Fenger, but found to-day that the specimen had been allowed to spoil. It was pedunculated, slightly adherent in places, trabeculated within, and quite full of collections of serum that coagulated upon exposure to air. The patient was operated upon two weeks ago, and is passing through a rapid and easy convalescence. The pedicle was treated extra-peritoneally, and the abdominal cavity closed without drainage.

The PRESIDENT exhibited

### A SUBSEROUS FIBRO-MYOMA OF THE CERVIX UTERI AND AN OVARIAN CYST.

I have here a subserous fibro-myoma of the cervix uteri and an ovarian cyst, which were removed three weeks ago from the same patient by vaginal section. I made first an exploratory incision in the recto-uterine cul-de-sac, and got behind the tumor, but could not get over it into the free peritoneal cavity. I then separated the uterus from the bladder, reached over the fundus, and ascertained the relation of the parts. I then pulled down the cervix and ligated the broad ligaments from below upwards. The capsule of the tumor was covered by a thin layer of peritoneum, except where it was imbedded in the cervical walls.

The interesting point was the size of the tumor, its relations, and the apparent impossibility of getting it out without taking the whole uterus. Although the operation was difficult, its severity did not seem great, for the patient is getting along very much the same as after a normal confinement.

### DERMOID CYSTS OF THE OVARY.

I have here a dermoid tumor consisting of two cysts removed five weeks ago. The tumor was about the size of a child's head and filled with chocolate-colored fluid and hairs. Some of the fluid escaped and flowed into the peritoneal wound. The peritoneal cavity was flushed with water and drained. The recovery was the same as a favorable case of oöphorectomy. The other ovary had undergone cystic degeneration.

SPECIMEN FROM TAIT'S OPERATION.

Here are four ovaries showing different stages of cystic degeneration. This pair was removed from a young girl who had been treated without benefit for the last three years. She was steadily losing ground. The diagnosis was ovaritis. They were removed about nine days ago.

Here is a pair removed four days ago. They commence to show the appearance of some of the larger tumors. The patient has been an invalid for seven years and was supposed to be losing her mind. Both are doing well.

